# Supplementary material for: Comparative Analysis of Cytokine Profiles in Cerebrospinal Fluid and Blood Serum in Patients with Acute and Subacute Spinal Cord Injury
Source: Biomedicines. 2023 Sep 26;11(10):2641. doi: 10.3390/biomedicines11102641 (PMC10604120; doi:10.3390/biomedicines11102641)
Supplement: Supplementary file 1 [file biomedicines-11-02641-s001.zip › Supplementary Table S1.pdf]

Table S1. Cytokine concentrations (ng/mL) in CSF and blood serum at 3, 7 and 14 days post-spinal cord injury in patients and uninjured control subjects.

Cytokine	CSF				Blood Serum		
	**Uninjured Control**	**3 dpi**	**7 dpi**	**14 dpi**	**Uninjured Control**	**3 dpi**	**7 dpi**
CCL26	1.83 (1.49)	26.53 (49.22)	12.71 (18.73)	29.89 (71.25)	2.00 (1.46)	14.87 (29.67)	9.49 (9.47)
	2.06 (0.44–3.06)	13.58 (2.95–20.28) *	7.98 (1.83–13.83) *	8.69 (1.83–18.32)	1.38 (0.93–2.46)	8.34 (3.77–13.77) **	7.97 (0.97–11.88) *
IL8	85.76 (58.74)	394.96 (608.69)	45.90 (74.71)	85.05 (176.30)	3.92 (3.91)	37.58 (171.92)	2.06 (6.13)
	7.23 (0.73–19.03)	49.05 (25.82–606.05) *	15.77 (0.46–40.11)	14.86 (3.39–42.92)	1.72 (0.69–7.43)	0.59 (0.05–1.34) *	0.64 (0.05–1.15) *
IL6	770.85 (308.25)	3299.32 (5184.97)	802.66 (2543.30)	385.09 (890.10)	6.54 (7.19)	4595.94 (21495.77)	6.15 (9.12)
	2.72 (0.34–7.47)	159.82 (20.36–6097) **	28.66 (0.87–275.19)	7.05 (5.10–66.34)	5.03 (2.45–5.61)	5.84 (3.32–11.32)	1.70 (0.22–7.33)
CCL23	3.79 (3.63)	58.16 (91.97)	33.85 (52.82)	31.76 (70.31)	74.37 (148.79)	114.60 (190.44)	474.01 (1991.08)
	2.54 (0.73–6.46)	21.05 (11.69–56.93) **	8.12 (2.40–27.35)	7.17 (1.61–11.04)	15.56 (6.23–77.32)	28.73 (11.06–168.27)	13.78 (7.39–122.53)
CCL22	13.67 (15.44)	54.74 (49.80)	25.78 (30.02)	84.57 (293.49)	10.41 (9.80)	36.36 (41.16)	42.83 (107.43)
	9.38 (3.39–14.78)	25.37 (15.43–88.21) *	16.30 (11.54–21.33)	12.44 (7.19–25.98)	7.10 (3.43–14.99)	15.31 (9.28–51.76)	16.63 (8.46–23.18)
IL1b	0.51 (0.44)	1.47 (2.04)	0.59 (0.64)	1.15 (2.12)	5.08 (1.67)	0.78 (0.62)	0.59 (0.64)
	0.18 (0.10–0.80)	0.71 (0.31–1.50)	0.47 (0.11–0.79)	0.68 (0.11–0.96)	5.33 (3.77–6.51)	0.76 (0.40–1.09) #	0.50 (0.02–0.88) #
CXCL9	42.46 (64.80)	22.73 (27.37)	12.52 (12.89)	48.43 (156.97)	114.44 (154.44)	14.99 (14.80)	15.48 (23.09)
	5.21 (2.18–59.83)	7.24 (3.62–35.97)	7.74 (2.65–19.68)	5.54 (2.33–13.18)	70.53 (40.30–136.71)	9.45 (3.28–24.02) #	6.57 (2.65–14.88) #
IFN-γ	11.31 (5.11)	17.21 (17.34)	12.60 (9.19)	17.10 (10.70)	0.42 (0.20)	10.78 (8.55)	9.40 (10.13)
	8.79 (3.71–11.77)	14.55 (3.92–25.39)	13.82 (8.26–16.27)	15.13 (8.50–24.89)	0.39 (0.24–0.60)	10.36 (3.90–16.69) #	5.76 (0.40–16.27) **
IL10	3.61 (3.32)	18.06 (31.71)	6.04 (9.52)	5.83 (7.82)	12.40 (7.81)	6.45 (17.49)	3.15 (5.28)
	1.73 (0.13–3.64)	9.59 (4.21–13.60)	2.74 (1.00–7.65)	2.91 (0.64–7.36)	12.14 (6.47–15.23)	2.98 (0.13–4.07) **	0.57 (0.13–3.70) #
CXCL6	10.16 (11.95)	10.61 (7.26)	6.66 (9.04)	31.12 (86.38)	2.61 (2.25)	7.62 (5.79)	10.31 (9.01)
	3.24 (0.73–18.61)	11.86 (5.68–13.90)	5.74 (0.73–7.47)	6.19 (2.70–9.41)	2.07 (0.80–3.11)	5.95 (4.32–10.59) **	8.75 (6.67–13.21) **
CXCL11	0.45 (0.49)	1.31 (1.27)	0.99 (1.11)	2.25 (4.29)	72.70 (175.50)	1.56 (2.41)	0.92 (1.33)
	0.35 (0.04–0.65)	0.93 (0.42–2.04)	0.77 (0.01–1.02)	0.74 (0.28–1.72)	2.02 (1.21–36.61)	0.68 (0.01–1.51) **	0.23 (0.01–1.51) **
IL4	3.44 (2.08)	6.27 (5.05)	3.37 (2.92)	5.14 (4.29)	1.58 (1.21)	5.00 (4.29)	6.08 (5.04)
	1.49 (0.58–5.03)	6.20 (2.56–9.03)	3.50 (0.15–4.87)	4.36 (2.35–5.99)	1.00 (0.82–2.26)	4.50 (3.81–5.11) *	4.86 (3.81–7.89) **
CCL7	16.75 (13.56)	57.68 (88.45)	63.76 (86.88)	37.07 (22.39)	10.28 (5.16)	27.90 (17.13)	26.32 (22.44)
	18.92 (2.59–28.49)	33.15 (12.75–58.73)	33.15 (24.61–62.72)	35.78 (27.26–51.57)	12.02 (6.15–13.56)	27.78 (15.86–37.12) **	20.38 (10.29–35.92) *
CCL3	1.13 (1.51)	4.40 (8.90)	2.77 (3.68)	3.52 (4.26)	3.45 (3.65)	1.80 (2.72)	1.66 (2.06)
	0.64 (0.11–1.30)	2.01 (0.72–3.27)	1.11 (0.74–3.65)	1.99 (1.15–3.43	2.19 (1.51–3.95)	0.99 (0.79–1.37) *	1.08 (0.46–1.69) *
CCL17	2.48 (2.61)	8.96 (10.43)	5.25 (5.82)	8.00 (13.07)	55.24 (82.44)	17.47 (22.41)	6.22 (9.66)
	1.04 (0.35–5.24)	2.02 (0.61–15.46)	1.27 (0.60–10.33)	1.50 (0.50–10.95)	25.96 (7.71–39.98)	5.10 (0.12–30.80)	0.08 (0.08–11.25) #
CCL8	1.84 (2.00)	21.46 (44.34)	10.40 (16.67)	37.70 (101.13)	22.55 (21.68)	8.84 (13.36)	6.76 (7.42)
	1.13 (0.26–2.86)	4.19 (2.00–9.58)	2.02 (1.10–13.12)	5.11 (0.97–12.44)	13.56 (5.74–34.23)	5.42 (3.73–8.80)	4.67 (1.18–9.77) **
CCL19	37.07 (42.37)	55.46 (46.23)	102.12 (150.85)	72.25 (62.93)	74.32 (64.38)	27.17 (30.54)	15.05 (21.11)
	25.28 (10.48–46.36)	46.69 (12.32–83.93)	57.89 (11.93–144.13)	61.54 (27.76–92.07)	67.44 (8.54–114.62)	15.84 (3.62–40.38)	3.39 (0.84–18.98) **
IL16	35.71 (16.60)	99.85 (160.59)	37.29 (35.50)	47.55 (63.34)	119.37 (154.81)	63.89 (103.36)	39.12 (99.61)
	15.86 (8.70–82.28)	31.05 (20.36–90.12)	27.14 (17.44–56.47)	24.76 (14.85–49.33)	71.38 (27.64–131.39)	25.20 (11.42–36.90)	15.25 (7.65–29.64) **
GMCSF	4.47 (11.94)	14.86 (17.74)	9.70 (10.92)	13.26 (15.60)	13.03 (8.07)	7.14 (7.80)	6.18 (10.36)
	0.45 (0.19–3.51)	8.37 (1.18–20.88)	4.18 (0.19–16.60)	3.81 (1.80–21.19)	8.81 (7.62–17.18)	5.70 (0.19–11.97)	0.42 (0.19–11.13) **

* Padj < 0.05, ** Padj < 0.01, and # Padj < 0.0001 comparing to uninjured control subjects.
